# Supplementary material for: The interactome of intact mitochondria by cross-linking mass spectrometry provides evidence for coexisting respiratory supercomplexes
Source: Mol Cell Proteomics. 2017 Dec 8;17(2):216–32. doi: 10.1074/mcp.RA117.000470 (PMC5795388; doi:10.1074/mcp.RA117.000470)
Supplement: Supplemental Data [file supp_RA117.000470_133922_0_supp_23978_fzffwf.zip › spectra_annotation/mito_spectra_annotation/138-1-4-1-12-1.pdf]

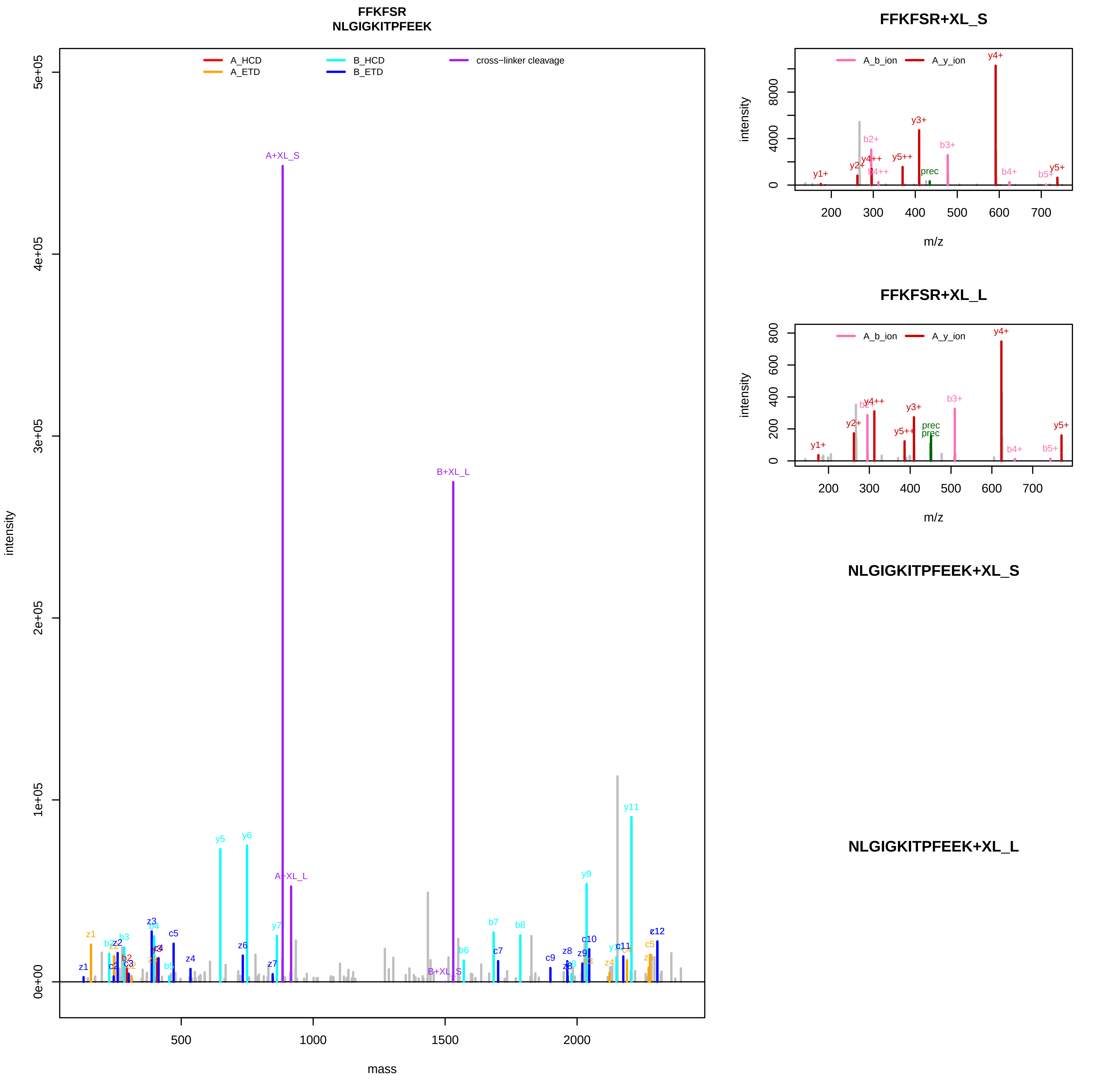
FFKFSR
NLGIGKITPFEEK
A_HCD
A_ETD
B_HCD
B_ETD
cross−linker cleavage
A+XL_S
B+XL_L
A+XL_L
B+XL_S
y5
y6
y7
y9
y11
b7
b8
c5
c7
c9
c10
c11
c12
z1
z3
z4
z6
z7
z8
z9
intensity
mass
0e+00
1e+05
2e+05
3e+05
4e+05
5e+05
500
1000
1500
2000
FFKFSR+XL_S
A_b_ion
A_y_ion
y1+
y2+
y3+
y4+
y4++
y5+
y5++
b2+
b3+
b4+
b4++
b5+
prec
intensity
m/z
0
4000
8000
200
300
400
500
600
700
FFKFSR+XL_L
A_b_ion
A_y_ion
intensity
m/z
0
200
400
600
800
NLGIGKITPFEEK+XL_S
NLGIGKITPFEEK+XL_L
